# The Fine Legacy of Giovanni P. Martelli (1935–2020), a Preeminent Plant Virologist and the Founder of Modern Grapevine Virology

**DOI:** 10.3390/v15010210

**Published:** 2023-01-12

**Authors:** Ahmed Hadidi, Marina Barba, Luisa Rubino

**Affiliations:** 1 U. S. Department of Agriculture, Agricultural Research Service, Beltsville, MD 20705, USA; 2 CREA-Research Center for Plant Protection and Certification, 00156 Rome, Italy; 3 National Research Council, Institute for Sustainable Plant Protection, 70126 Bari, Italy

Giovanni Paolo Martelli passed away on 8 January 2020 [1], nine days short of his 85th birthday. Giovanni was a brilliant scientist of great integrity and honor. He was born in Palermo, Sicily, Italy, on 17 January 1935. After attending high school in Bari, Puglia (Apulia), Italy, he enrolled at the University of Bari, from which he graduated cum laude in Agricultural Sciences in 1956. In 1957, he joined the University of Catania, Sicily, Italy, for eight months as a Voluntary Assistant of Plant Pathology and a Fellow of CNR (Consiglio Nazionale delle Ricerche [the National Research Council]). He joined the University of Bari in 1958 as a Volunteer Assistant Plant Pathologist and rose through the professional ranks, achieving the rank of Full Professor in 1973 [2]. In 1975, Giovanni and Ahmed first met during Giovanni’s one-day visit to the Plant Virology Laboratory, U.S. Department of Agriculture, Beltsville, Maryland, and their friendship flourished for 45 years.

At the beginning of his scientific career, Giovanni had acquired highly significant knowledge of Plant Pathology. In the 1950s, he investigated the biology and control of plant pathogenic fungi that infect olive trees, grapevine, fruit and vegetable crops, and described new micromycetes and diseases [2]. At a young age, he immediately realized the importance of international exchange and collaboration for scientific and cultural advancement. As a result, Giovanni spent sabbaticals at the Institute of Botany, University of Liverpool, England, UK (1960); the Department of Plant Pathology, University of California (UC), Davis, CA, USA (1961–1963); and the Scottish Crop Research Institute, Invergowrie, Dundee, Scotland, UK (1964). His sabbatical at UC, Davis represented a scientific turning point: working under the supervision of the internationally renowned Dr. William B. Hewitt, who is known as the father of grapevine virology, Giovanni gained knowledge and experience in grapevine viruses and virus diseases and developed great interest in plant virology [2]. In 1958, Dr. Hewitt discovered nematode transmission of the soil-borne grapevine fanleaf virus [3]. Consequently, at Davis, Giovanni also studied agricultural nematology, and was among the initiators of research on the Italian nematode family *Longidoridae*. Upon his return to the Institute of Plant Pathology at Bari in 1963, Giovanni established the Plant Virology Laboratory to study viruses and virus diseases of economically important vegetable plants, as well as grapevines and fruit trees such as citrus, stone fruit, olive and fig. The Plant Virology Laboratory quickly became nationally and internationally eminent under Giovanni’s leadership.

Most of Giovanni’s teaching career was spent at the University of Bari. From 1965 to 1969, he taught Microbiology; he then spent a two-year term (1971–1973) teaching Plant Pathology at the University of Palermo and directing the Institute of the same name. Back at the University of Bari, he was appointed Full Professor and served as Chair of Plant Virology for 30 years, from 1980 to 2010, when he retired [1].

At the University of Bari, Giovanni directed the Institute of Plant Pathology (later to become a Department) from 1980 to 1986 and the Department of Plant Protection and Applied Microbiology from 2000 to 2006. His primary aim was always the advancement of knowledge, and he transmitted his passion to colleagues and collaborators. Among the faculty members involved in plant virology research when Giovanni was Chair of Plant Virology were Maria Antonietta Castellano, Donato Gallitelli, Pasquale Piazzolla, Antonio Quacquarelli, Marcello Russo, Vito Savino and Makis Vovlas. All held important academic and research positions during their careers.

As a member of the Board of Professors for the Ph.D. program in Plant Pathology from 1984 to 1991, and for the Ph.D. program in Plant Protection starting in 1992, Giovanni was an outstanding example of scientific integrity and rigor.



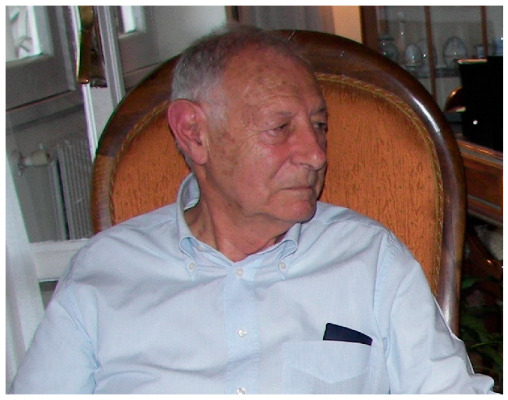

Giovanni Martelli in Rome in June 2012 relaxing with old friends.

His competence was widely acknowledged. Starting in 1972, Giovanni was a member of the Scientific Council of the Institute of Applied Phyto-Virology of the CNR in Turin. He was also a member of the Scientific Council of the Plant Biosynthesis Institute of the CNR, Milan.

In 1982, he established the CNR Study Center on Viruses and Virus-like Diseases of Mediterranean Crops, based in Bari, and became its director until his retirement in 2010. Among the scientists who were hired early by CNR and contributed to its scientific success were Donato Boscia, Angelantonio Minafra and Luisa Rubino. The Center no longer exists as such; it later merged with the Institute for Sustainable Plant Protection (CNR-IPSP), with Donato Boscia as its research director. From 1989 until its disbandment, Giovanni chaired the Scientific Council of the Research Group of the CNR on Viruses and Virus Diseases of Plants.

In 1985, Giovanni was among the supporters of the International Course on Production and Protection of Mediterranean Fruit Crops (now Integrated Pest Management of Mediterranean Fruit Crops) at the Mediterranean Agronomic Institute of Valenzano (Bari), an Italian subsidiary of Center International de Hautes Etudes Agronomiques Mediterraneennes, of which he was the coordinator of the related “Master of Science” program in Plant Virology until 2008.

Giovanni investigated viruses and virus diseases of crops grown in the Mediterranean basin, including fruit trees, grapevine [4], vegetable crops [5,6] and figs [7,8,9,10], among others. He identified and characterized over 30 new plant viruses. He was also interested in the study of virus–host interactions at the fine structural level. He conducted a series of studies on the cytopathology of viral infections [11,12], the structure and nature of inclusion bodies [13], cellular sites of viral nucleic acids and protein synthesis [14] and sites of intracellular accumulation of viral particles [7,15,16,17]. He was also interested in virus epidemiology, particularly that of viruses transmitted through the soil by nematodes [18,19,20], closteroviruses transmitted by pseudococcid scale insects [21,22] and viruses transmitted through seed [23,24,25] and pollen [24].

In the mid-1980s, Giovanni established a molecular biology/immunology research unit for the use of recombinant DNA technology and monoclonal/polyclonal antibodies in different research projects [2]. Some results of this research led to the production of transgenic plants which expressed various types of viral genes, some of which conferred resistance to infection. Moreover, viral RNAs were used as carriers of foreign genes; and the roles of extra-genomic molecules such as plant viral satellites and interfering defective RNAs were investigated.

Giovanni was an international recognized authority on viruses and virus diseases of grapevine, and he is considered the founder of modern grapevine virology [26,27,28,29]. Plant Virologists world-wide agree that Giovanni and his group in Bari transformed and advanced the science of grapevine virology from the 1960s to its current 21st Century high standard, which is based on the state of the art in genomics, molecular biology, immunology and electron microscopy. Giovanni shared this tremendous progress in grapevine virology with scientists from Europe, the USA, Canada, South America, North Africa, the Middle East, Japan, China, Australia and New Zealand. Giovanni was President of the International Council for the Study of Viruses and Virus Diseases of Grapevines (ICVG) for more than 30 years, from 1987 to 2018, and he kindly made Dr. William B. Hewitt the Honorary President of this group, as a sign of his gratitude.

Giovanni never ignored or forgot the application of applied plant virology to grapevine and other plant species. In 1963, he established a thermotherapy unit for producing virus-free grapevine and fruit trees [30]. In the mid-1980s, he organized an in vitro tissue culture unit for the restoration of grapevine and other economically important plant species. Virus elimination, combined with selection of healthy plants in the field, allowed the establishment of successful certification programs for grapevine and fruit trees in Italy [31]. Giovanni was also one of the first to establish nuclear, foundation and mother stock plants for breeding of certified clones of rootstocks of *Vitis* and *Prunus* spp. [32].

Giovanni carried out study missions on behalf of the Food and Agricultural Organization (FAO) of the United Nations, United Nations Development Program (UNDP), CNR, and the Italian Ministry of Foreign Affairs. He was a member of the Steering Committee of the FAO-UNDP Regional Project on Control of Virus and Virus-like Diseases of Fruit Crops, the Comitè d’Evaluation of the Institut National de la Recherche Agronomique (INRA) and the Comitè Scientifique du Laboratorie d’Ingenierie Agronomique de l’ENSAT-INPT. Giovanni was also invited to participate in study missions by officials from five countries in North Africa (Morocco, Algeria, Tunisia, Libya and Egypt), eight countries in the Middle East (Cyprus, Israel, Lebanon, Jordan, Syria, Turkey, the United Arab Emirates and Yemen), most Eastern European countries, one country in Central Asia (Afghanistan), two countries in the Far East (China and Japan), Australia and New Zealand. These missions established international scientific collaborations with scientists in various institutions in these countries.



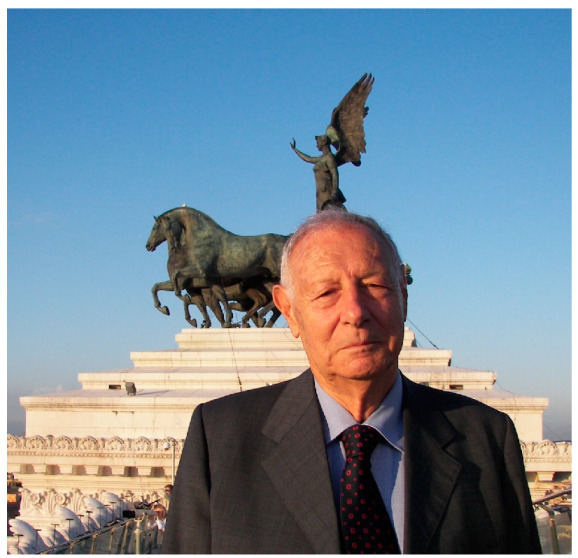

Giovanni Martelli in June 2012 on top of Vittorio Emanuele II Monument (Il Vittoriano) in Rome City Center. Behind Giovanni, the Winged Victory Statue on Chariot.

Giovanni had an excellent background in Latin. In 1978, he became an active member of the International Committee on Taxonomy of Viruses (ICTV) of the International Union of Microbiological Societies. He chaired the study group on the family *Closteroviridae* [33,34]. He was a member of the study groups on the families *Flexiviridae* [35,36], *Tombusviridae* [37], *Tymoviridae* [38] and *Fimoviridae,* which includes the genus *Emaravirus* [39]. For seven years, from 1987 to 1993, Giovanni chaired the ICTV Plant Virus Subcommittee. He promoted the application of plant viral taxonomy based on family–genus–species, which was already adopted by animal, fungal and bacterial virologists. In fact, up to the early 1990s, plant viruses were clustered into “groups” of individual viruses sharing major biological features. However, *groups* and *viruses* were not recognized taxa and did not correspond to any taxon in the “parallel” classification. Group members did not represent species and had only vernacular English names [40,41]. The coexistence of two different taxonomic systems became less tenable as soon as the acceptance of the concept of species gained momentum. The increasing amount of molecular data demonstrated that, besides mutations, reassortment and recombination—two mechanisms by which species may arise—also occur with plant viruses, supporting the reconsideration of the plant virus taxonomy system aimed at a new classification scheme. This was the first step towards the alignment of plant virus taxonomy to other virus taxonomies. Giovanni understood the importance of a unified virus taxonomy, regardless of the infected host. He played an active role in this process by implementing the transformation of some groups into families or genera, and by grouping genera into families. Giovanni was particularly proud of this part of his scientific career, and he used to smile remembering that such a great amount of work was shared with colleagues by regular mail, since e-mail was not commonly used yet. On the other hand, he was deeply aware of the potential of the molecular data for disclosing similarities among viruses and accelerating virus taxonomy development considering phylogeny and evolution. This vision is now reality: the former five-rank virus taxonomy (species, genus, subfamily, family, order) was recently extended to a 15-rank classification hierarchy in line with the Linnean taxonomic systems [42].

Giovanni described novel plant virus genera, e.g., *Foveavirus*, *Aureusvirus*, *Tepovirus* [43,44,45,46]. Moreover, Giovanni contributed significantly to the taxonomy of several other plant viral genera, families and orders. He continued serving on the ICTV Executive Committee as an elected member. In 1999, in recognition of his dedicated contribution to virus taxonomy, Giovanni was elected a “Life Member” of the ICTV [1]. In 2021, the ICTV acknowledged his outstanding contributions to Plant Virology and Virus Taxonomy by ratifying a plant virus order carrying his last name: *Martellivirales* [47], including seven families: *Bromoviridae*, *Closteroviridae*, *Endornaviridae*, *Kitaviridae*, *Mayoviridae*, *Togaviridae* and *Virgaviridae*.

Giovanni served as an associate editor of *Phytopathology*, *European Journal of Plant Pathology* and *Vitis*. He also served as senior editor and, from 2002, as Editor-in-Chief of the *Journal of Plant Pathology* (Formerly, *Rivista di Patologia Vegetale*). He was extremely convinced of the importance of editorial roles in the advancement of knowledge, and his reviews were always very helpful to the authors, allowing them to improve their research work and presentation of results.

Giovanni was Chairman of the International Working Group on Vegetable Viruses of the International Society of Horticultural Sciences from 1979 to 1981, and President of the Italian Phytopathological Association from 1980 to 1986. He also represented the Italian Phytopathological Association in the International Society for Plant Pathology. Moreover, he was a member of the Executive Boards of the Italian Society of Virology and of the Italian Society of Plant Pathology, of which he was President (2002–2004). Giovanni was a member of many scientific societies and committees, including the Italian Academy of Vine and Wine (of which he was a member of the Academic Council), a member of the National Winemaking Committee of the Ministry for Agricultural and Forestry Policies, the Georgofili Academy, the National Academy of Oil and Olive, the National Academy of Agriculture and Apulian Academy of Sciences, and he was a corresponding member of the National Academy of the Lincei and of the Agricultural Academy of Turin. He was a member of the American Phytopathological Society, the Association of Applied Biologists, the British Society of Plant Pathologists, the Mediterranean Phytopathological Union, the Society of Plant Molecular Biology, the Italian Plant Protection Society the Italian Society of Plant Pathology and the Italian Society of Virology. Giovanni also chaired numerous scientific meeting sessions at national and international conferences.

Giovanni is the co-author of a textbook on Plant Virology and one book on viruses of vegetable crops. He is also the co-author of two books [26,29] and two directories [27,28] on grapevine virology. The latest comprehensive book on grapevine virology, published in 2017, [29] was co-edited by Giovanni. It contained 33 chapters, and he contributed some of these chapters as the sole author. In addition, he is the co-author of about fifty book chapters and several scientific articles in virology encyclopedias (Springer Index of Viruses, The Atlas of Plant Viruses, Encyclopedia of Virology) and non-virology encyclopedia (Enciclopedia Agraria Italiana, Encyclopedia of Life Sciences). He wrote about 600 scientific reports, of which about 300 research articles were published in refereed international journals of high standing.

In recognition of his scientific achievements, Giovanni was awarded the Gold Medal Diploma from the Académie d’Agriculture de France for a book he co-authored on grapevine viruses in 1982; and in 1997, he was elected Fellow of the American Phytopathological Society [2]. Moreover, in 1999, he was awarded the F. Maseri-Florio World Prize for Distinguished Research in Agriculture for his outstanding contribution to our knowledge on grapevine viruses and virus diseases; in the same year, he was named a “Life Member” of the ICTV. In 2006, he was appointed an Honorary Professor at Huazhong Agricultural University, Wuhan, China. In 2007, he was awarded the Antico Fattore Award of the Georgofili Academy, and in 2008, he was appointed an Honorary Member of the Italian Academy of Vine and Wine. In 2009, he received the Pioneer in Virology Award from the Italian Society of Virology; in 2010, he was appointed an Honorary Member of the Italian Society of Plant Pathology.

During the last decade, Giovanni vigorously defended the integrity of science and scientists when plant pathologists at the CNR in Bari were viciously and falsely attacked by individuals or groups of individuals deprived of phytopathological scientific knowledge while Bari scientists correctly identified the bacterium *Xylella fastidiosa* subsp. *pauca* as the causal agent of the olive trees decline in southern Italy, completely sequenced the bacterial DNA genome and determined its size as a 2,514,616 base pair, and established that the bacterial country of origin is Costa Rica in Central America [48,49]. The spittlebug *Philaenus spumarius* was experimentally proven to be the vector of the olive-infecting bacterial strain [50].

Beyond his scientific achievements, Giovanni’s legacy lives on in the hundreds of people across the world that he trained, mentored, encouraged and supported in various ways. He advised many graduate students and postdoctoral fellows, who have gone on to careers in academia, industry and beyond. Giovanni was generous in accepting seminar invitations and delivering keynote addresses at national and international meetings. As such, thousands of junior and established scientists had the opportunity to interact with him throughout his career. His interactions were characterized by warmth and generosity. Overall, Giovanni will be remembered as a brilliant plant virologist, the founder of modern grapevine virology and a very kind and wonderful human being who had a great impact on the people around him.

Giovanni is survived by his wife Nicoletta, their daughter Maria Carla and their son Giuseppe, and he was a proud grandfather to his five lovely grandchildren, whom he adored.
